# Four-Channel Ultrasonic Sensor for Bulk Liquid and Biochemical Surface Interrogation

**DOI:** 10.3390/bios14020066

**Published:** 2024-01-26

**Authors:** Donatas Pelenis, Dovydas Barauskas, Mindaugas Dzikaras, Darius Viržonis

**Affiliations:** Panevėžys Faculty of Technology and Business, Kaunas University of Technology, 37164 Panevėžys, Lithuania; donatas.pelenis@ktu.lt (D.P.); dovydasbarauskasacademic@gmail.com (D.B.); mindaugasdzikarasacademic@gmail.com (M.D.)

**Keywords:** CMUT, biosensor, SAW, BAW, ultrasound signal processing

## Abstract

Custom electronics tailored for ultrasonic applications with four ultrasonic transmit-receive channels and a nominal 25 MHz single channel frequency were developed for ultrasound BAW and SAW biosensor uses. The designed integrated microcontroller, supported by Python with a SciPy library, and the developed system measured the time of flight (TOF) and other wave properties to characterize the acoustic properties of a bulk of the liquid in a microchannel or acoustic properties of biological species attached to an analytic surface in real time. The system can utilize both piezoelectric and capacitive micromachined ultrasound transducers. The device demonstrated a linear response to changes in water salinity. This response was primarily attributed to the time-of-flight (TOF) changes related to the varying solution density. Furthermore, real-time DNA oligonucleotide-based interactions between oligonucleotides immobilized on the device’s analytical area and oligonucleotides attached to gold nanoparticles (Au NPs) in the solution were demonstrated. The biological interaction led to an exponential decrease in the acoustic interfacial wave propagating across the interface between the solution and the solid surface of the sensor, the TOF signal. This decrease was attributed to the increase in the effective density of the solution in the vicinity of the sensor’s analytical area, as Au NPs modified by oligonucleotides were binding to the analytical area. The utilization of Au NPs in oligonucleotide surface binding yields a considerably stronger sensor signal than previously observed in earlier CMUT-based TOF biosensor prototypes.

## 1. Introduction

The ability to miniaturize the sensing devices through modern fabrication techniques and integrate microfluidic environments on top of them allows for a complete lab-on-a-chip (LOC) building. Such LOC systems unlock quick, cheap, and easy-to-use diagnostics for various ailments available for patients without physical medical staff intervention and can be performed at home.

The ultrasonic time-of-flight (TOF) measurement technique is a non-destructive method that can be used for distance sensing as well as for measuring the elasticity and density of materials. The technique involves sending an ultrasonic pulse through a material and measuring the time it takes for the pulse to travel through the material [1,2,3,4,5]. This technique can also be used for attenuation measurement, though the accuracy potential is less than that of other ultrasonic techniques [6]. While the surface binding of proteins and DNA changes the elasticity and viscosity of the interfacial media, it will also change the time-of-flight signal [1,7,8,9]. However, TOF changes due to the biomolecule binding process would be marginally small due to the similarity of the density and elasticity of the biomolecules and the solution in which the process is happening. Direct detection would require an extremely high resolution of TOF measurement, which is achievable only if acoustic waves with more than 1 GHz frequency are used. This is technically challenging and expensive. Therefore, acoustic biosensing often utilizes the binding of additional heavy mass entities—typically gold nanoparticles (Au NPs)—together with the biomolecules of interest onto the sensing layer [10,11].

Acoustic TOF signal measurements for biological entity detection are relatively under-researched, yet many other methods using acoustics have been demonstrated. A common way of classifying acoustic biosensors is bulk acoustic wave (BAW) and surface acoustic wave (SAW) devices. While BAW devices, which utilize the propagation of the acoustic waves through the bulk of the liquid sample and, therefore, are simpler to implement, SAW devices are particularly attractive for biosensing applications since they allow the immobilization of biomolecules of interest onto the bioreceptor sensing layer and quantify their concentration via specific changes in a selected parameter of acoustical wave [12]. Various SAW-based biosensor concepts have already been demonstrated [13]. An aptamer-based leaky surface acoustic wave (LSAW) biosensor has been shown to detect MCF-7 breast cancer cells down to 32 cells/mL with minimal response to two other types of cells [14]. A Lamb wave-based DNA biosensor for the detection of bacterial meningitis with a detection limit of 84 mg/µL was developed using ZnO thin films [15].

Besides the piezoelectric SAW devices, microelectromechanical system (MEMS)-based devices have been shown to be a suitable basis for film bulk acoustic resonator (FBAR) and SAW-type biosensor construction [16]. Even though these devices are considered MEMS, they still utilize piezoelectric materials to generate the waves. One type of mechanical MEMS system is microcantilevers. A magnetically and optically actuated microcantilever array-based biosensor was demonstrated for the detection of Hepatitis A and C viruses [17]. However, the need for the integration of an optical subsystem and an electric coil for data readout makes such systems relatively unwieldy. Erdil et al. demonstrated a low-cost disposable cantilever-based system for the detection of aflatoxin M1 (AFM1) [18]. The designed system is capable of detecting a minimum of 14 µg of AFM1 with a limit of detection of 4.63 µg. These values are too high to be transferred to biochemical molecule detection applications. Furthermore, it relies on arduous sample preparation techniques and specific magnetic nanoparticles, is not integrable with microfluidics, and is a single-time-use device. Additionally, a cantilever-based MEMS device was designed for the detection of the SARS-CoV-2 virus [19]. The proposal was based on a piezoelectric material PZT-5A, and the device was only simulated not built. Another proposed piezoelectric cantilever-based biosensor was designed for the detection of heavy metal ions, such as manganese, lead, copper, and cadmium, in water samples [20]. Again, the device was not built, only simulated, it relied on an additional container, holding the cantilever, and the transducing functionalization layer was not defined. Other simulation-based MEMS biosensors were also proposed [21,22,23,24].

Electrochemical MEMS-based biosensors were also manufactured and demonstrated [25,26]. They utilize a carbonized version of photoresist SU8 for the detection of lactic acid and a cancer biomarker platelet-derived growth factor-BB. However, these devices use typical MEMS production methods for the device geometry formation but do not utilize any mechanical motion in the biosensor working principle.

Additionally, quartz crystal microbalance-based MEMS biosensors were also demonstrated for the detection of an inflammation biomarker C-reactive protein and immunoglobulin G [27,28], with detection limits of 1 ng/mL for both of the biomolecules.

The majority of other microcantilever-based biosensors work in gaseous environments, detecting specific illness-related volatile organic compounds or other molecules, since typically liquid environments exhibit too much cantilever vibration dampening to make them useful [29]. A specific MEMS-based device that generates acoustic waves mechanically instead of piezoelectrically—a capacitive micromachined ultrasound transducer (CMUT) was demonstrated to have some particular advantages in biosensing [30]. It was recently shown by our group that bioanalytes can be detected by the measurement of the transverse Scholte-type wave propagation delay between two CMUT interdigital transducers by using convolutional neural networks for signal processing [31]. Since this type of signal processing imposes the intensive use of hardware resources, the need for dedicated electronics is recognized. In this paper, the concept, design, and testing of a dedicated TOF electronics system in the context of BAW and SAW modes of operation is presented.

## 2. Materials and Methods

### 2.1. The Concept and Structure of the Single PCB TOF Electronics

The time delay or TOF measurement of an interfacial acoustic wave utilizes the pitch-catch acoustic measurement, where a short acoustic pulse is emitted by the sending transducer and later captured by the receiving transducer. Due to the extensive signal processing requirements, oversampling of the received acoustic signal was chosen. Working frequency band of the sensor electronics was limited by the capability of a high-speed low-power 8-bit analog to digital converter (ADC), providing 250 megasamples per second conversion rate. As the data-processing algorithm required at least 5 times the oversampling beyond the Nyquist criterion (10 samples per period instead of 2 are needed), the single-channel nominal carrying frequency of the acoustic signal can be up to 25 MHz, while the actual operating frequency is determined by the design of ultrasound transducers, number of simultaneously used channels, and the measurement algorithm. Another limitation is the real-time memory capacity of the chosen microcontroller. Therefore, a Raspberry Pi 4 microcomputer was selected, providing adequate memory to capture up to 100 microseconds of the received signal at maximum sampling frequency.

**Figure 1:**
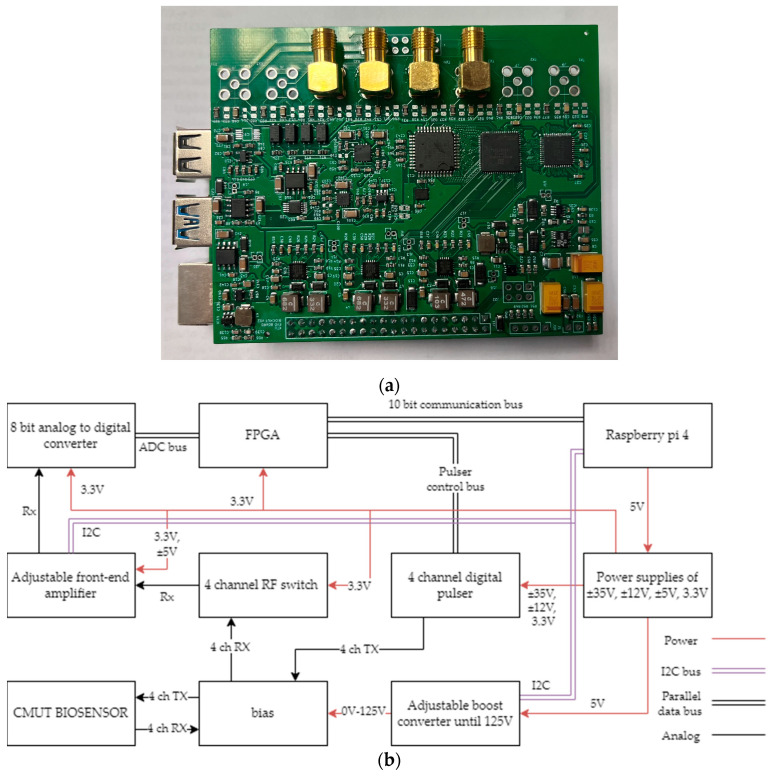
Sensor electronics: (**a**) overall view of the PCB; (**b**) functional diagram.

The single-board electronics are illustrated in Figure 1. Only two of four channels (two connectors for each transmit/receive phase) were employed in the work described within this paper. The next two channels are dedicated as reference channels for calibration and elimination of non-informative factors. Since the current work was tailored towards proof of concept, we did not engage in calibration procedures. The receiver input can be switched between up to 4 ultrasound transducer channels by a radio frequency switch. Further, at the receiver side, the cascade of 20 dB low-noise and adjustable gain (up to 10 dB) amplifiers perform the preliminary signal conditioning of the received signal before passing it to an ADC. The digital 8-bit signal is buffered by a field-programmable grid array (FPGA) and then fetched by a microcontroller and stored in the microcontroller’s memory.

Sensor excitation (transmission) signal is synthesized by the FPGA, according to the specification determined by the microcontroller’s code. A four-channel digital pulser, driven by the FPGA, directly outputs bipolar ±35 V pulses to up to 4 transmitting ultrasound transducers. The frequency and the burst length of each excitation signal can be controlled separately.

The rest of the electronics is dedicated to satisfying the power requirements of the appropriate elements. The 5 V power supply provided by the microcontroller is used to power several DC-to-DC energy converters. There is a step-down converter to provide 3.3 V for logic circuits and four boost converters to maintain bipolar power supplies of ±5.5 V, ±5 V, ±12 V, and ±35 V, supplemented by linear regulators for analog components. There is also a boost converter providing 0 V–125 V regulated DC voltage for CMUT bias.

### 2.2. Signal Processing and Analysis

The microcontroller controls and synchronizations transmit and receive circuits. The digital signal processing and measurement algorithm can be custom-designed and flexibly adapted since Python with SciPy library utilization is supported. During the testing and experiments described in this paper, a simple pulse amplitude tracking algorithm was used [32]. As the digital signal processing algorithm can operate outside of the real-time process, the practical limitations of the algorithm complexity are only due to the available program memory.

### 2.3. CMUT-Based TOF Measurement Channel

**Figure 2:**
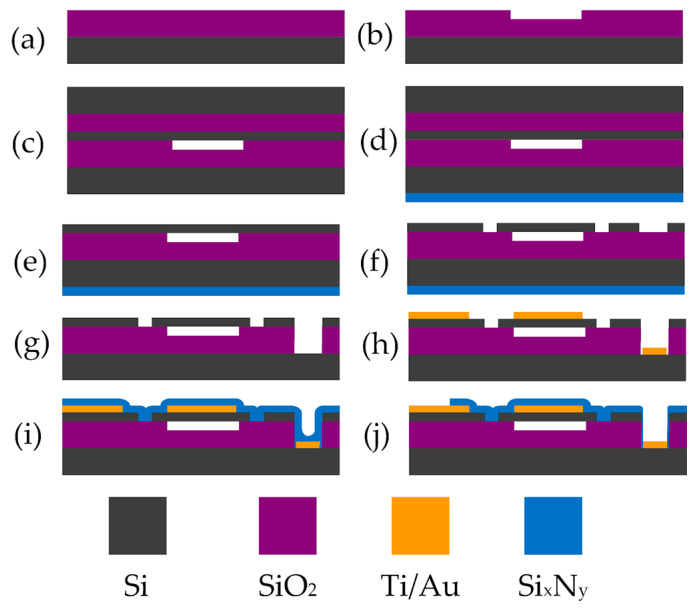
Main microfabrication steps of CMUTs: (**a**) thermal oxidation; (**b**) formation of vacuum cavities; (**c**) wafer bonding; (**d**) protective silicon nitride layer deposition; (**e**) removal of the carrier wafer; (**f**) deep reactive ion etching; (**g**) opening of the bottom electrode; (**h**) formation of the top electrode and metallization of contact pads; (**i**) formation of a protective silicon nitride layer; (**j**) RIE for top and bottom electrode contact pad openings.

The production of capacitive micromachined ultrasonic transducers involved wafer bonding technology of silicon wafers. In the initial step, thermal oxidation was performed on the highly doped (less than 0.01 Ωcm) background 4-inch silicon wafers to deposit a 300 nm thick silicon dioxide (SiO_2_) layer. CMUT cavity pattern was defined on the silicon dioxide surface by the lithography process, followed by etching in a buffered oxide etch (BOE) solution (Figure 2a,b). The depth of these cavities, defining the vacuum gap size of the electrostatic structure, was controlled by the etching time. In the subsequent step, silicon wafers were fusion bonded to silicon-on-insulator (SOI) wafers with a 1 ± 0.5 μm thick monocrystalline silicon device layer separated from the handle wafer by a 2000 nm thick buried oxide layer (BOX, Figure 2c). In the following step, the protecting 250 nm thick silicon nitride film (Figure 2d) was deposited, and then handle wafer was removed by chemical–mechanical polishing (CMP) and wet etching in a heated tetramethylammonium hydroxide (TMAH) solution (Figure 2e), thus releasing the membranes. In the second photolithography step, pattern for structure separation was defined, and deep reactive ion etch (DRIE) was performed (Figure 2f). In the third photolithography step, openings for the bottom ground electrode were created, and the backside protective silicon nitride layer was removed (Figure 2g). The fourth lithography step employed the lift-off process, facilitating the metallization of the top electrodes to increase the conductivity. The metal stack consisted of 25 nm titanium, 100 nm copper, and 75 nm gold. In the fifth lithography step, the metallization of the contact pads was carried out, also employing the lift-off process (Figure 2h). A passivation silicon nitride layer, protecting the surface conductors from short-circuiting by the conductive liquids or other external factors, was formed by plasma-enhanced chemical vapor deposition (PECVD) (Figure 2i). Finally, the last photolithography step was performed to open contact pads using reactive ion etching (RIE) (Figure 2j).

**Figure 3:**
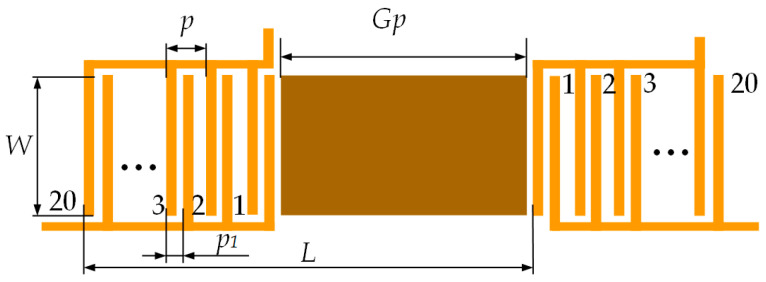
Interdigital CMUT biosensor layout.

An interdigital CMUT device has been used during the experiments with interfacial waves. Arranging of the finger pairs enabled two-phase excitation and reception of the acoustic waves, as described in many earlier publications [30,31,33]. The layout of the interdigital CMUT device pair is shown in Figure 3. Here, p is the IDT period—distance between finger pairs—p1 is distance between sub-finger pairs, W is the finger length or aperture width, Gp is the length of the square area of the biological interaction site, and L is the distance between the transmitter and the receiver. Detailed parameters of the microfabricated IDT CMUT device are given in Table 1.

**Figure 4:**
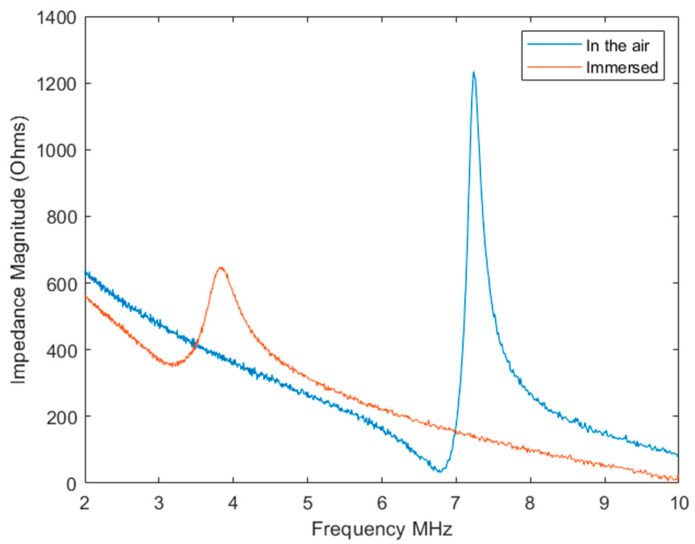
Impedance magnitude spectra of the single CMUT interdigital element while operating in the air and in immersion.

The Agilent 4395A network analyzer was employed for the bare CMUT chip testing and served as a reference channel for measuring frequency spectra in the one-port impedance analyzer mode. The 86 V bias voltage was maintained by the Agilent N5752A. Measured impedance magnitude spectra for the single IDT channel of the CMUT in the air and immersion are shown in Figure 4. It can be seen that CMUT exhibits resonance close to 7.5 MHz when operated in air and nearly 3.8 MHz when immersed in water.

### 2.4. Interfacial Acoustic Wave Measurement Setup

**Figure 5:**
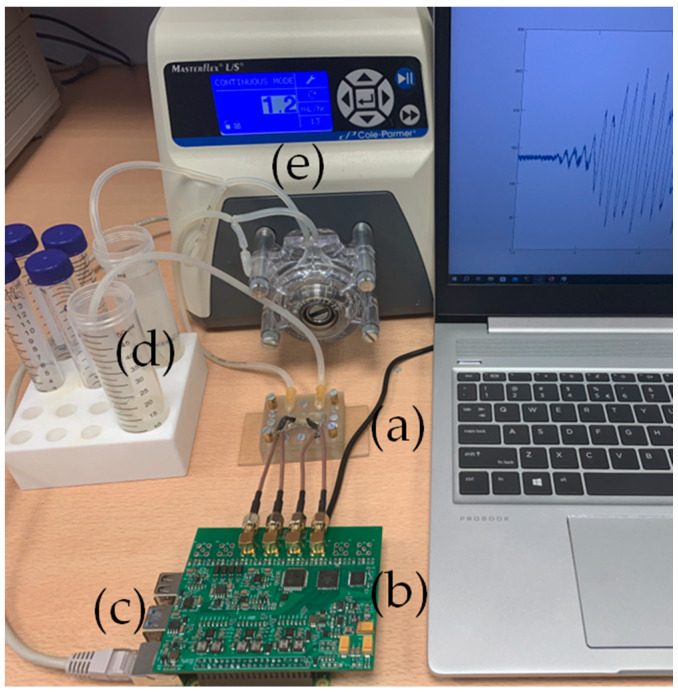
Experimental setup for interfacial waves delays experiment in a microchannel: (**a**) microfluidic chamber with CMUT chip and microchannel; (**b**) electronics board; (**c**) Raspberry Pi 4 microcomputer ethernet connection to a PC; (**d**) reservoirs with analytes and tubing; (**e**) a peristaltic pump.

**Figure 6:**
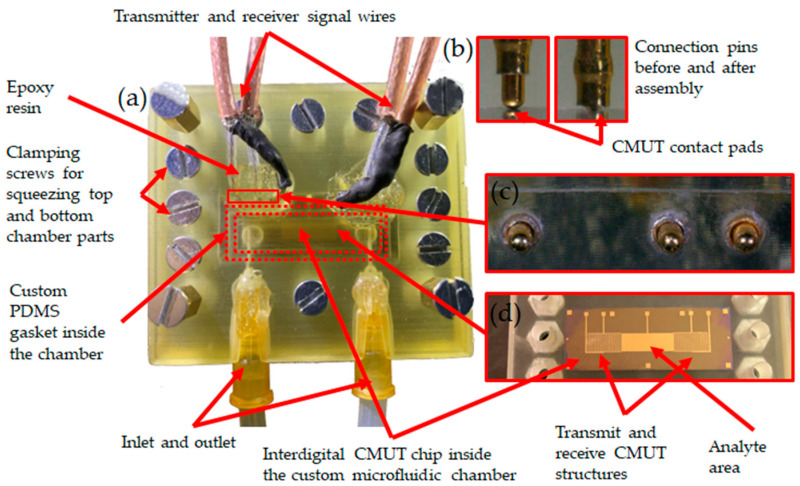
Detailed illustration of the microfluidic chamber used during experiments: (**a**) the top part of the chamber is visible, and the bottom is used as a surface for assembly and to maintain a water-tight seal between the top part of the chamber and CMUT surface; the microchannel is directly above the CMUT interdigital transmit and receive structures with the gold analyte area in between; (**b**) connection pins before and after clamping the two sides of the microfluidic chamber; (**c**) pins for connections to top and bottom electrode of the CMUT contact pads friction fitted into the chamber walls; (**d**) interdigital CMUT chip when top of the microfluidic chamber is open; transmit and receive structures with the gold analyte area in between are visible.

**Figure 7:**
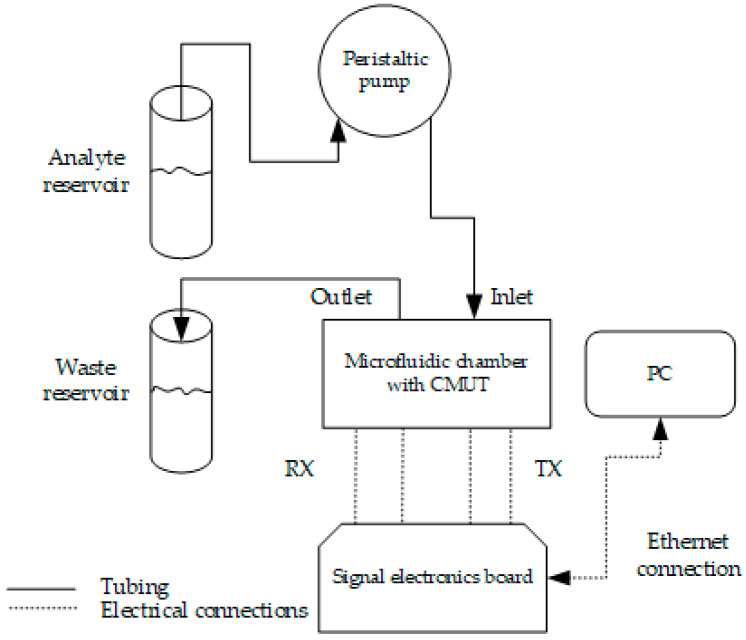
The experimental setup for interfacial waves delays experiment in a microchannel.

Since the surface immobilization of the biological samples and analytes is the main method of acoustic biosensing [13,14,31], we continued testing the electronics by connecting them with the CMUT chip (see Figure 5 and Figure 6). The experimental setup for the interfacial waves delays experiment consisted of a custom 3D-printed microfluidic chamber, analyte, and waste reservoirs, a peristaltic pump for pumping the analyte into the microchannel, a custom signal generation and acquisition electronics board with Raspberry Pi microcomputer, and a laptop with custom MatLab software for data analysis. Figure 7 shows the schematic diagram illustrating the main connections between the elements of the experimental setup, while Figure 5 shows the real-life setup. Figure 6 is a more detailed illustration of the microfluidic chamber. The chamber was 3D-printed using stereolithographic Anycubic printer with UV-clear photosensitive resin. The dimensions of the microfluidic chamber and the microchannel are given in Table 2. The two parts of the chamber, the top and bottom, had tightening screws positioned around the microchannel casing edges. Inlet and outlet ports were positioned upwards from the microchannel ends (see Figure 5a). The water-tight seal was manufactured from a custom PDMS gasket placed between the top of the microfluidic chamber and the surface of the CMUT chip (Figure 5a). The electrical connections were established by spring pins friction-fitted in the microfluidic chamber walls directly contacting the contact pads on the IDT CMUT chip (see Figure 5b,c). Figure 5b shows the connection pins before and after clamping the two sides of the microfluidic chamber. Figure 5d shows the interdigital CMUT chip when the top of the microfluidic chamber is open, and transmit and receive structures with the gold analyte area in between are visible.

### 2.5. Au NPs and Oligonucleotides

Au NPs, 20 nm in diameter, stabilized with citrate buffer (Sigma Aldrich, St. Louis, MO, USA), were functionalized with thiolated oligonucleotides for subsequent binding onto gold surface of the custom-made CMUT-based biosensor in the microfluidic chamber, as described in Figure 6. DNA oligonucleotides (IDT) were custom-synthesized with such sequences: 5′-ATGGCAACTATACGCGCTAG-3′ (linker 1), 5′-AAACGACTCTAGCGCGTATA-3′ (linker 2), 5′-AAGTCAGTTATACGCGCTAG-3′ (non-matching linker 1), 5′-ACACTAAACTAGCGCGTATA-3′ (non-matching linker 2), 5′-AGTCGTTT/3ThioMC3-D/-3′ (3’-thiolated 1), 5′-GTTGCCAT/3ThioMC3-D/(3′-thiolated 2). “/3ThioMC3-D/” denotes 3′ modification of a protected thiolic group on a 6-carbon alkane spacer. Linker 1 and linker 2 oligonucleotides are partially complementary with each other. Additionally, 3′-thiolated 1 oligonucleotide is fully complementary to linker 1, and 3′-thiolated 2 oligonucleotide is fully complementary to linker 2. The non-matching linkers 1 and 2 are complementary to thiolated oligonucleotides in the same fashion, but they are not complementary to each other. Before use, the thiolated oligonucleotides were deprotected using TCEP (Sigma Aldrich) by adding 100× TCEP concentration to the oligonucleotide solution suspended in TE buffer and incubating for 2 h at room temperature. One set of 100 µL of Au NPs was functionalized with the deprotected 3′-thiolated 1 oligonucleotides, and a separate set of 100 µL of Au NPs was functionalized with the deprotected 3′-thiolated 2 oligonucleotides.

Two sets of Au NPs functionalized with either 3′-thiolated 1 or 3′-thiolated 2 oligonucleotides were mixed together. Separately, either matching linkers 1 and 2 were mixed together, or non-matching linkers 1 and 2 were mixed together and incubated for 10 min. Finally, either the matching or non-matching linkers were introduced into the Au NP mixture, mixed with the pipette, and 2 µL of the mixture was deposited onto the Nanodrop one C spectrophotometer pedestal. The measurements were started immediately, and sample absorption was recorded every 5 s for 180 s for each sample.

### 2.6. SAW Change by Au NP Surface Binding through DNA Oligonucleotide Interaction

The gold analyte area on the CMUT transceiver chip was functionalized with 3′-thiolated 1 oligonucleotide by introducing into the microfluidic chamber 2 mL 500 nM oligonucleotides suspended in 1xTE buffer. The system circulated the oligonucleotides through the chamber for 10 min using the peristaltic pump. After that, the surface and the system were washed with a 1× TE buffer twice using the same method for 10 min each time. Afterward, either hybridized linker 1 and linker 2 oligonucleotides or non-matching set of linker 1 and linker 2 were introduced to the system to bind to the 3′-thiolated 1 oligonucleotides already present on the gold analyte surface and circulated in the system for 10 min. The system was again washed twice with a 1× TE buffer. Finally, a set of Au NPs functionalized with 3′-thiolated 2 oligonucleotides were prepared, as described in the previous section, introduced into the system via the same method, and circulated for 10 min.

### 2.7. The Bulk Acoustic Wave Experiment

**Figure 8:**
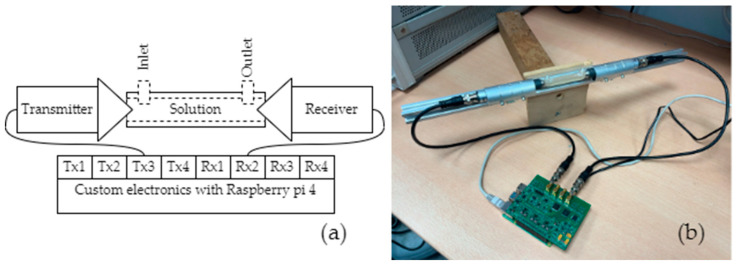
Testing of the electronics by a bulk acoustic wave propagation experiment: (**a**) schematic representation of an experiment; (**b**) photo of an experimental setup.

**Figure 9:**
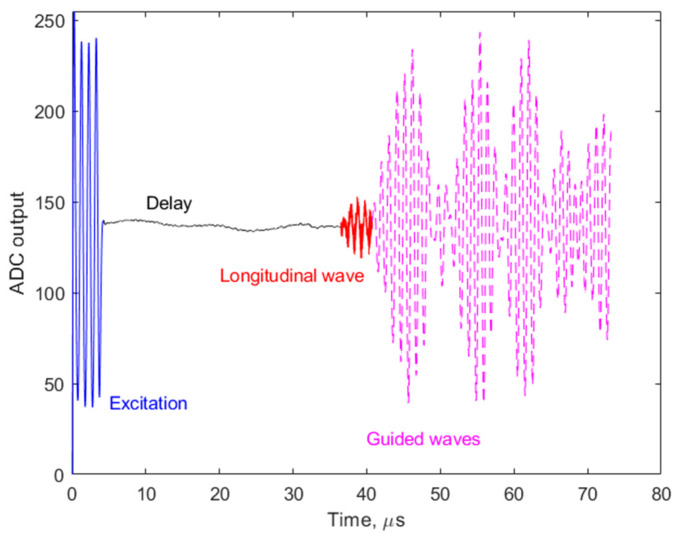
Single frame of longitudinal wave TOF measurement.

**Figure 10:**
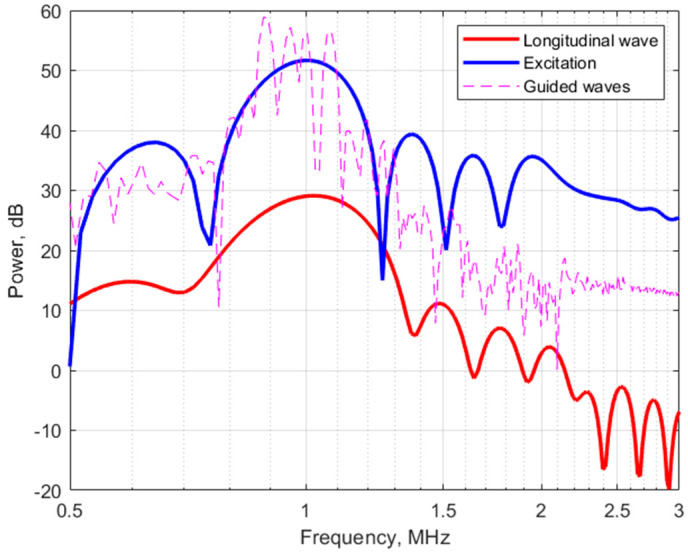
Power frequency spectrum plot of the received ultrasonic pulse. The spectrum was calculated using the formula power_spectrum_dB_b = 10 × log10(power_spectrum_b); where power_spectrum_b = (1/N) × abs(X_b(1:N/2 + 1))^2^; where N is the number of elements in the data array and X_b—the output of a fast Fourier transform function, obtained from ADC output N data.

**Figure 11:**
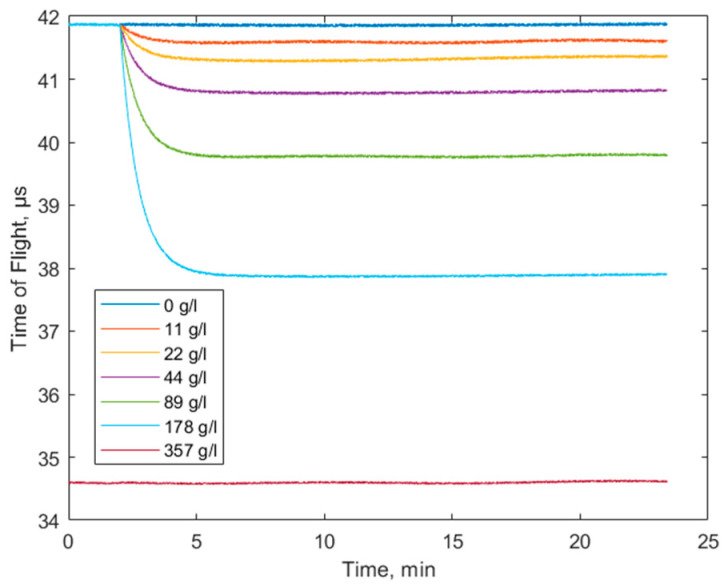
TOF monitoring of the water and NaCl mixing process. The concentration values in a legend correspond to the final concentrations after the mixing is complete.

Initial testing of the TOF algorithm was performed by monitoring the bulk acoustic waves during the water/saline mixing process. A cylindrical 62 mm length cell with 2.5 mm inner diameter was fabricated to fit the piezoelectric ultrasound transducers with conical concentrators, designed for 1 MHz operation frequency, as shown in Figure 8. The entire volume of the liquid in this case was 300 μL. A single frame of the TOF measurement is shown in the Results section (Figure 9 in the time domain and Figure 10 in the frequency domain). This frame was captured during the steady-state phase of the saline mixing experiment with 178 g/L (see also Figure 11 in the Results section).

## 3. Results

### 3.1. The Bulk Acoustic Wave Experiments

The longitudinal acoustic wave traveling through the bulk of the water has the earliest time of arrival, while the rest of the received waves with larger amplitudes are attributable to the non-informative, highly dispersive waveguide modes, related to the multiple reflections of the acoustic waves from the sidewalls of the measurement cell and corresponding interferences. In the frequency domain, one can note a slight high-frequency shift of the bulk longitudinal wave power spectrum with respect to the power spectrum of the excitation signal, which can be the result of interactions between the ultrasonic waves and the propagation medium. However, this phenomenon was not explored any further in this work. The power spectrum of the guided modes is noisy, but it can still be identified as approximately centered at the same frequency as the excitation.

Since the concentration of a NaCl solution is related linearly to the mass density of the solution (at constant temperature), the relationship between the concentration and the speed of sound is also linear. This establishes a reliable and easily verifiable testbed. The cell had two ports for liquid introduction and removal. The experiment was carried out in two steps. In the first step, 150 mL of distilled water was introduced, monitoring the initial TOF isoline. After two minutes, 150 μL of saline solution with a particular NaCl concentration was introduced, and a decrease in the TOF signal, reflecting a gradual increase in the solution density, was observed. The mixing was driven solely by the diffusion process, i.e., no active agitation, except for the ultrasonic waves themselves, was used. After the mixing process was complete (after approximately the fifth minute of the experiment), the final solution concentration had half of the initial saline concentration. An exception to the aforementioned protocol is the case of the fully saturated saline solution, where the entire volume of the cell was filled with the 357 g/L NaCl solution to have the bottom line of the measurable TOF value. The results of this experiment are illustrated in Figure 11.

**Figure 12:**
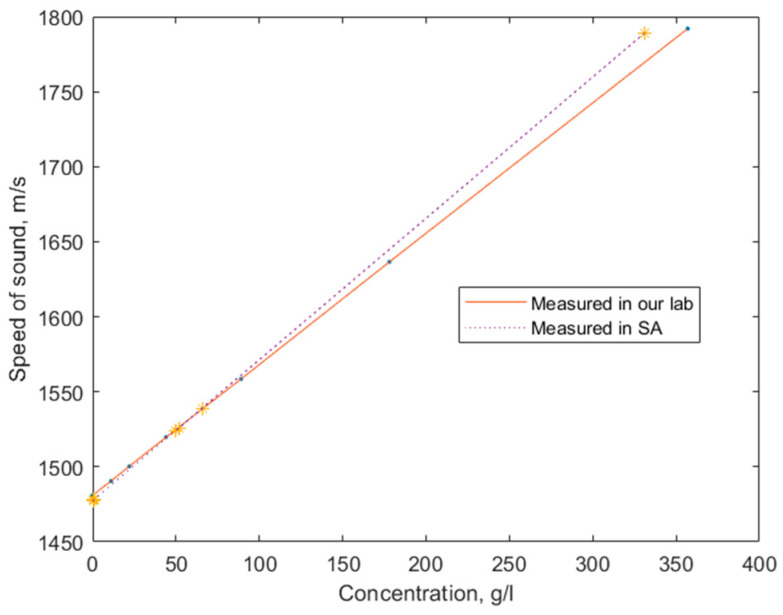
Comparison of the steady-state ultrasound speed in different saline concentrations measured during our experiment and during earlier experiments described in [34].

For the cross-verification of the measurement results, the steady-state TOF values were compared to the earlier published saline concentration and speed of sound data [34,35]. For an adequate comparison, we converted our measured steady-state TOF values to the speed of sound values, using 62 mm as the travel distance. The comparison is illustrated in Figure 12. The slight discrepancy of the measured values at high concentrations can be attributed to uncontrolled factors, such as temperature or precise composition of the saline solutions.

**Figure 13:**
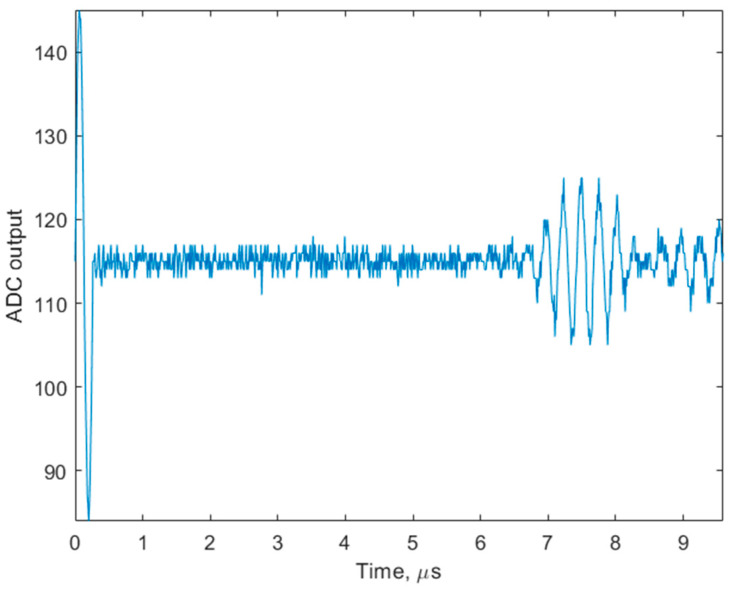
Single frame of the transverse wave TOF measurement.

Testing was continued by connecting the measurement cell, containing the CMUT chip and the microchannel (Figure 7). The transverse acoustic waves were excited by emitting a single period, 10 Vpp pulses of 3.8 MHz with the transmitting IDT and received by the receiving IDT within the same chip. The bias voltage was kept at 85 V for both transmit and receive transducers. The distance between both IDTs through the analytical area was 10 mm (see Figure 3 and Table 1). The single frame of the TOF measurement is illustrated in Figure 13. It was captured while the microchannel was filled with distilled water, and the analytical area had no modification. Assuming the rated distance of 0.01 m between the IDTs and the first measured zero-crossing at 6.76 μs, the measured speed of the transverse wave was calculated to be 1479 m/s. This corresponds well to the data available from the previous research [30,33]. Here, the application of CMUTs has specific benefits because of their ability to produce short pulse excitations. While shortening excitation pulses will decrease the amplitude of the received signal, it will also create the potential for better definition.

### 3.2. DNA Oligonucleotide Interactions

**Figure 14:**
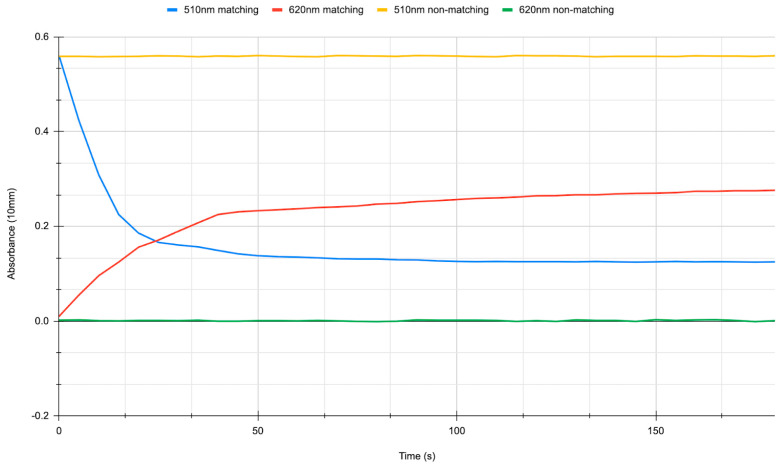
Spectrophotometry results for cross-linking thiolated oligonucleotide-coated Au NPs via DNA oligonucleotides. Blue line indicates absorption at 510 nm wavelength, and red line indicates absorption at 620 nm when matching linker oligonucleotides are introduced into the two set thiolated oligonucleotide-coated Au NP solution. Yellow line indicates absorption at 510 nm wavelength, and green line indicates absorption at 620 nm when non-matching linker oligonucleotides are introduced into the two-set thiolated oligonucleotide-coated Au NP solution.

The biochemical interaction of DNA oligonucleotide hybridization was tested by reacting thiolated oligonucleotide-coated Au NPs with linker oligonucleotides, as described in the Materials and Methods section. The results are depicted in Figure 14. The matching linker oligonucleotides would hybridize with thiolated oligonucleotide-coated Au NPs, bringing NPs together, crosslinking, and changing the average sphere size in Mie scattering and, therefore, light absorption at specific wavelengths. Initially, non-crosslinked 20 nm size Au NPs have the highest absorption value at 510 nm. When the matching oligonucleotides are introduced, they crosslink the Au NPs, bringing them together and changing the absorption. This manifests as decreased absorption at the initial 510 nm wavelength (blue line values in the figure) and increased absorption at a selected 620 nm wavelength (red line values). Considering the crosslinked NPs come in different sizes, the shifted absorption peak from 510 nm to higher values becomes broader, yet 620 nm was chosen for monitoring as it was the highest value. If, however, the experiment is repeated with non-matching linker oligonucleotides introduced instead of the linker oligonucleotides, no change in absorption over time is observed for either 510 nm wavelength (yellow line) or 620 nm (green line), indicating a stable NP suspension and no hybridization of the complementary non-matching linker oligonucleotides. This result, taken in contrast with the previous linker oligonucleotide experiment, indicates that the Au NP crosslinking and the subsequent absorption change due to NPs coming into proximity is oligonucleotide induced and not due to any other factors.

**Figure 15:**
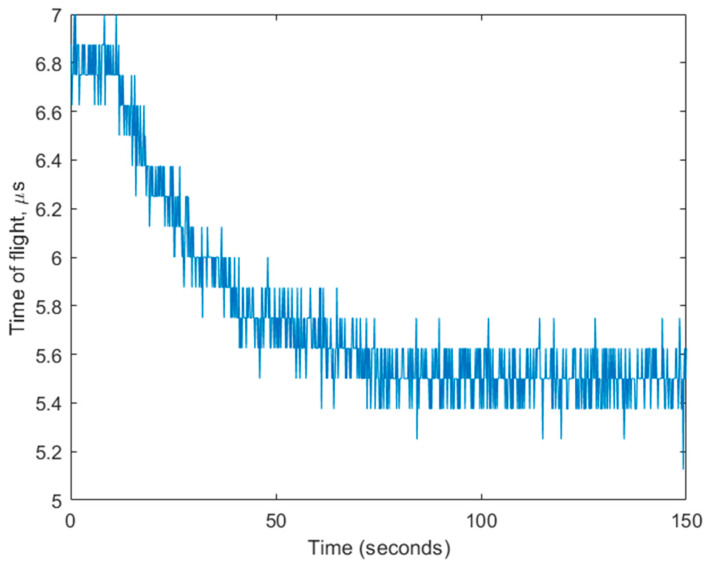
Unprocessed (raw) time of flight readings during the immobilization of thiolated oligonucleotide-coated Au NPs over the analytical surface coated by DNA oligonucleotides.

With Au NPs and the oligonucleotide biochemical interaction system established, the experiment was transferred to the microfluidic chamber with one of the sets of Au NPs replaced by the CMUT chip’s gold analyte area, as described in the Materials and Methods section. The setup was used to detect changes in the TOF signal when observing SAWs. The data are given in Figure 15. The TOF signal was observed for 150 s. The signal decreases from 6.8 µs to 5.5 µs (a difference of 1.1 µs) on the full saturation of Au NP immobilization onto the surface via oligonucleotide hybridization. The curve reaches saturation levels in 70 s. The noise level is relatively high, yet manageable for the detection of bound analytes, because the TOF measurement resolution, which is 80 ns in this case (relative to 25 MHz sampling frequency), is sufficient. As a reference to our previous research [31], resolution improvement can be achieved if more sophistication is put into signal processing. Notably, in the current work, only the simple tracking of the envelope of the received pulse for the signal processing was used. There, bovine serum albumin protein was adsorbed onto the surface non-specifically and yielded a 16 ns TOF difference, while the neural network algorithm was involved in the signal processing. The discrepancy in signal strength is attributable to the use of Au NPs in this research, as they increase the average medium density above the gold surface and, therefore, improve the signal.

## 4. Discussion and Conclusions

The designed custom electronics and the accompanying microfluidic devices have demonstrated the ability to interrogate liquid bulk properties as well as its use in SAW biosensor applications with the ability to measure propagation delay (time-of-flight—TOF) of the acoustic signal and potentially other properties of acoustic waves since the received signal is oversampled at a high resolution. The four-channel version demonstrated an 80 ns TOF resolution. However, the resolution can be enhanced with higher sampling frequencies, reaching up to 250 MHz for the single-channel configuration. The electronics are compatible with various types of ultrasound transducers and can be easily adjusted for advanced acoustic analysis with corresponding firmware modifications. The four-channel transmitter is controlled by an FPGA capable of synthesizing a wide range of frequencies and excitation patterns. Additionally, the integrated microcontroller can efficiently run relatively complex data processing algorithms over the data received from those four channels. The TOF response was linear with a sensitivity of at least 11 g/L NaCl concentration in the bulk wave propagation delay experiment. The DNA oligonucleotide interactions immobilizing the Au NPs onto the surface demonstrated a significant response in the TOF signal, indicating its use for biochemical interactions. The utilization of heavy Au NPs improves the biochemical signal in the CMUT-based biosensor.

The proposed approach of acoustic wave propagation delay measurement in biosensing stands out from conventional biosensing methods and applications, which are mostly related to the frequency and phase responses [36]. The popularity of these methods is due to the marginally small changes to the acoustically important properties, such as the mass density, viscosity, and elasticity (compressibility) of analyte solutions during specific biological interactions. For example [37], phase loop locking systems are used to detect resonance changes during biotin-streptavidin binding, and recent reviews of acoustic wave measurements in biosensing applications [38] indicate only a few cases where the attenuation and phase delay of the transmitted acoustic waves are measured. Additionally, the full control over the transmit pattern and oversampling of the received acoustic waves is often considered to be an overkill for compact and low-priced devices (as biosensors are supposed to be) since electronics for transmit wave synthesis and received wave sampling are considered pricey and energy demanding. Most of the research on acoustic biosensing is related to the development of sensing principles and is still dependent on the use of expensive laboratory equipment [15,16,39,40,41]. In the current work, the concept of stand-alone, single-board, miniature, and low-cost electronics was demonstrated as a proof of concept for future acoustic wave biosensors, eliminating functional complexity and providing highly flexible opportunities for the biosensor user.

By simplifying functionality and offering unparalleled flexibility of use, demonstrated advancement will move the development of acoustic biosensors to a new step in the future. Particularly noteworthy is the compact, multichannel nature, which has significant potential for the enhancement of the efficiency and accuracy in detecting and identifying DNA of viruses and infectious agents. Multiple boards can be combined within one detection system for the parallel processing of many samples for more complex DNA analysis, ensuring robustness and lowering costs.

## Figures and Tables

**Table 1:** Microfabricated IDT CMUT device parameters.

Size	Name of the Parameter
80 µm	Single membrane length
30 µm	Single membrane width
200 µm	IDT period (“pitch”), p
50 µm	Distance between sub-fingers, p_1_
3 mm	Aperture width, W
10 mm	Biological interaction site width, Gp
13.7 mm	Distance between transmitter and receiver, L
20	Number of finger pairs
7.5 MHz	Resonance frequency

**Table 2:** Dimensions of the microfluidic chamber and the microchannel.

Size	Name of the Parameter
80 µm	Chamber width
30 µm	Chamber length
200 µm	Chamber height
50 µm	Microchannel width
3 mm	Microchannel length
10 mm	Microchannel height

## Data Availability

The data presented in this study are available upon request from the corresponding authors.
